# Design Knowledge for Deep-Learning-Enabled Image-Based Decision Support Systems: Evidence From Power Line Maintenance Decision-Making

**DOI:** 10.1007/s12599-022-00745-z

**Published:** 2022-04-01

**Authors:** Julius Peter Landwehr, Niklas Kühl, Jannis Walk, Mario Gnädig

**Affiliations:** 1 Institute of Information Systems and Marketing (IISM) / Karlsruhe Service Research Institute (KSRI), Kaiserstraße 89, 76133 Karlsruhe, Germany; 2 Netze BW GmbH, Schelmenwasenstraße 15, 70567 Stuttgart, Germany

**Keywords:** Decision support system, Design science research, Computer vision, Infrastructure inspection and maintenance, Power line, Deep learning

## Abstract

With the ever-increasing societal dependence on electricity, one of the critical tasks in power supply is maintaining the power line infrastructure. In the process of making informed, cost-effective, and timely decisions, maintenance engineers must rely on human-created, heterogeneous, structured, and also largely unstructured information. The maturing research on vision-based power line inspection driven by advancements in deep learning offers first possibilities to move towards more holistic, automated, and safe decision-making. However, (current) research focuses solely on the extraction of information rather than its implementation in decision-making processes. The paper addresses this shortcoming by designing, instantiating, and evaluating a holistic deep-learning-enabled image-based decision support system artifact for power line maintenance at a German distribution system operator in southern Germany. Following the design science research paradigm, two main components of the artifact are designed: A deep-learning-based model component responsible for automatic fault detection of power line parts as well as a user-oriented interface responsible for presenting the captured information in a way that enables more informed decisions. As a basis for both components, preliminary design requirements are derived from literature and the application field. Drawing on justificatory knowledge from deep learning as well as decision support systems, tentative design principles are derived. Based on these design principles, a prototype of the artifact is implemented that allows for rigorous evaluation of the design knowledge in multiple evaluation episodes, covering different angles. Through a technical experiment the technical novelty of the artifact’s capability to capture selected faults (regarding insulators and safety pins) in unmanned aerial vehicle (UAV)-captured image data (model component) is validated. Subsequent interviews, surveys, and workshops in a natural environment confirm the usefulness of the model as well as the user interface component. The evaluation provides evidence that (1) the image processing approach manages to address the gap of power line component inspection and (2) that the proposed holistic design knowledge for image-based decision support systems enables more informed decision-making. The paper therefore contributes to research and practice in three ways. First, the technical feasibility to detect certain maintenance-intensive parts of power lines with the help of unique UAV image data is shown. Second, the distribution system operators’ specific problem is solved by supporting decisions in maintenance with the proposed image-based decision support system. Third, precise design knowledge for image-based decision support systems is formulated that can inform future system designs of a similar nature.

**Supplementary Information:**

The online version contains supplementary material available at 10.1007/s12599-022-00745-z.

## Introduction

With modern-day societies increasingly relying on electrical power, the importance of continuous electricity supply cannot be overlooked. Continuous power supply has two central building blocks – the electricity generation as well as its transmission and distribution to the consumer. From the perspective of transmission or distribution system operators, the maintenance program of the power line infrastructure is crucial in avoiding unexpected disruptions. These system operators have typically adopted condition-based maintenance programs to minimize the probability of equipment breakdowns (Pagnano et al. 2013; Jalil et al. 2019). Condition-based maintenance is considered as a three-step process of data acquisition, data processing, and maintenance decision-making (Jardine et al. 2006), with the last step integrally including maintenance order planning (Gopalakrishnan et al. 2015).

Assessing the condition of the components in an electricity network includes inspecting towers or poles with their connected components, conducting power lines, and the surrounding vegetation of the two previous elements, as it can cause short circuits. Operators routinely examine these aspects regarding faults. Based on the operator’s composed inspection reports, maintenance engineers need to compile situation-dependent, well-defined, complete, and prioritized maintenance orders. This requires the consideration of several other factors, such as infrastructure topology, available workforce and skill sets, scheduled infrastructure revision projects, and bundling of maintenance orders. Fast and accurate inspection as well as complete and exhaustive data and information dissemination are crucial for efficient maintenance decision-making and can reduce the risk of power outages due to component failures, increasing the reliability of electricity supply.

Traditionally, the inspection is performed through human visual observation by means of manual ground inspection, helicopter-based patrolling, and tower climbing. These inspection methods are costly, time consuming, partly hazardous, do not comprehensively capture data, and are hardly scalable. Recent technical advances in the fields of unmanned aerial vehicles and image processing or computer vision^1^ have spurred the development of automated power line inspection. Specifically, deep learning has proven to boost the performance of image processing applications (LeCun et al. 2015) – converging towards human level performance or even surpassing humans (He et al. 2015b). Researchers are therefore increasingly focusing on the automatic vision-based detection of components and the immediate diagnosis of faults in the inspection of power lines (Liu et al. 2020) leaving only the eventual maintenance decision-making for human handling.

Previous research on power line maintenance has been scattered, focusing on the technical building blocks. Today, the majority of studies either focus on performing unmanned aerial vehicle inspection flights autonomously (Hui et al. 2018) (data acquisition), on task-specific image processing approaches for component detection and fault diagnosis (Nguyen et al. 2018) (data processing), or on orchestrating the various technical components (Huang et al. 2018; Homma et al. 2017) (interplay between data acquisition and processing). So far, little effort has been devoted to holistic and end-to-end considerations establishing a relationship between the solely technical problems of automating the data acquisition and processing and the need for integrating and transferring the acquired data and extracted information into maintenance decision-making. To this end, we conduct a project to design and evaluate a suitable decision support system following the design science research paradigm (Hevner 2007) and its common research guidelines (March and Smith 1995; Winter 2008). We address the ever-increasing need for maintaining the impeccable condition of power lines, and consequently the reliability of electricity supply. We do so by utilizing available technological possibilities for holistic vision-based applications to provide decision support in scoping and planning maintenance orders for maintenance engineers through improved data and information quality. We focus on addressing this need by answering the following research question (RQ):

How can an automated, efficient, and useful vision-based power line maintenance decision support system be designed?

By answering this question, we unlock the still largely unregarded and nascent problem class of *image-based decision support systems*, which we believe to be the higher level abstraction for our specific vision-based power line maintenance decision support system. In particular, following the dual mission of design science research of developing usable artifacts for practice and generating theoretical knowledge for the knowledge base (Gregor and Jones 2007), we initially explore the challenges and issues of power line maintenance to derive a number of design requirements for *image-based decision support systems*. Subsequently, we conceptualize design principles based on justificatory knowledge from image processing and deep learning. Based on these design principles, we obtain a number of design features to be used as our application domain specific design for the *image-based decision support system* for vision-based maintenance of power line components. We instantiate these design features into a concrete artifact that allows us to rigorously evaluate the proposed design knowledge in practice.

The remainder of this work is structured as follows: Sect. 2 summarizes the existing relevant literature. Next, in Sect. 3, we introduce the research methodology. In Sect. 4, we conceptualize our design knowledge for *image-based decision support systems*, before we introduce the developed artifact as well as its various evaluations in Sect. 5. Finally, in Sect. 6, we discuss our research findings, reflect on the limitations of our work, and provide an outlook for future studies.

## Related Work

To determine the potential of extensively captured images of power line components (PLCs), we review related work and the literature background in several fields. First, we briefly introduce foundations regarding deep learning (DL) Sect. 2.1. In Sect. 2.2, we present how computer vision (CV) is used for infrastructure inspection in different application domains. Subsequently, in Sect. 2.3, we present related work regarding automated vision-based power line inspection using UAV-captured images. Afterwards, in Sect. 2.4, we examine image-based decision support systems (IB-DSS) as a way to harness images in efficient decision-making. We conclude this section by synthesizing the presented literature and depicting our research gap in Sect. 2.5.

### Deep Learning

Within the past decade, machine learning has shown significant results solving complex problems – both in theory as well as in application within industry (Brynjolfsson and Mcafee 2017). Especially in the field of DL,^2^ a family of algorithms solely based on artificial neural networks with multiple hidden layers, the developments grew rapidly (Bharati and Pramanik 2020).

DL overcomes a general limitation of machine learning to handcraft appropriate features in order to find and learn patterns in input data. The advanced architecture gives DL the capability to automate feature learning and consequently reduce human effort (Janiesch et al. 2021). Hence, DL is able to better deal with large-scale, noisy, and unstructured data.

The exact amount and size of layers is a design choice such that ideal architecture for a given problem and its data must be found through experimentation (Goodfellow et al. 2016). Each layer is subject to learning and computes non-linear input-output mappings which enables a DL model to represent extremely intricate functions of its input (LeCun et al. 2015).

Due to these capabilities, DL has brought breakthroughs in processing images, videos and audio like speech (LeCun et al. 2015). In particular, Convolutional Neural Networks (CNNs) a class of DL algorithms which excel at learning hierarchical features (Janiesch et al. 2021), are especially suited for the application to feature-rich data – like images. Therefore, DL is a promising candidate for applications within the field of CV.

### Computer Vision-Based Infrastructure Inspection

CV aims to equip computers with visual perception skills similar to the human ones (Szeliski 2010). CV models based on DL have led to a significant increase in performance – DL models have even been proven to surpass human-level performance for specific applications (He et al. 2015b). Typically, four different CV tasks are distinguished on static images (Griebel et al. 2019): in image *classification* the whole image is assigned a class label. Object *detection* additionally outputs an approximate location of the object of interest. *Semantic segmentation* produces even more fine-granular information, as each pixel is assigned a class label. In the specific case of *instance segmentation*, neighboring objects of the same class are distinguished additionally.

In the past years, several specific architectures have been developed to allow for these different CV tasks. While the two main optimization criteria are the accuracy of the prediction and the time inferred to obtain the solution ever more tailored solutions building on CNNs are being developed recently. Architectures such as VGG16 (Simonyan and Zisserman 2015) and ResNet (He et al. 2015a) for image classification and Faster R-CNN (Ren et al. 2015) and SSD (Liu et al. 2016) for object detection have proven to provide good accuracy at resonable inference time.

CV is utilized for infrastructure inspection in many application domains. The typical challenges addressed with CV in this area are cases where large amounts of physical objects are to be inspected and they are geographically remote and / or dispersed. Selected research articles are presented in the following and summarized in Table 1.

**Table 1:** Deep-learning-based power line inspection approaches to detect and diagnose multiple components and similar approaches from other application domains

Articles	Application domain	Component detection		Fault diagnosis		Design focus
		Components	Method	Components	Method	
Chatterjee et al. (2018)	Road surface inspection	Road	Graph-based hierarchical clustering	Road cracks	Multiple machine learning classifiers	$\checkmark$
Shihavuddin et al. (2019)	Wind turbine blade inspection	Leading edge erosion, vortex generator panel, vortex general panel with missing teeth, lightning receptor	Faster R-CNN	*Fault diagnosis* *treated as* *detection task*		x
Wei et al. (2019)	Railway track inspection	Railway track fastener	Faster R-CNN	*Fault diagnosis* *treated as* *detection task*		x
Zhu et al. (2018)	Powerline inspection	Spacer, bird nest, insulator, damper, tower plate, tower	Cascaded Faster R-CNN	–	–	x
Nguyen et al. (2019)	Powerline inspection	Pole, cross-arm, insulators	Cascaded Faster R-CNN/SSD/Yolo	Insulator, pole, top cap, cross-arm	ResNet50	x
Liang et al. (2020)	Powerline inspection	Defect tower foundation, insulator, grading ring, contact terminal, triple-plate, earth wire, bird thorn, bird nest, foreign body	Faster R-CNN	*Fault diagnosis* *treated as* *detection task*		x
Our work	Powerline inspection	Insulator, bird nest, fitting, safety pin	Cascaded Faster R-CNN/SSD	Safety pin	ResNet50	$\checkmark$

A major application area is road surface inspection and maintenance. Roads in bad condition can ultimately result in more accidents and higher costs (Baladi et al. 2017; Gleave et al. 2014). Thus, CV is utilized to automatically assess road surface condition and derive necessary maintenance actions. Over the last years this became possible without expensive, specialized hardware (compare, e.g., Quintana et al. 2016). Chatterjee et al. (2018) show how machine learning-based CV can be used to detect road surface cracks and develop a “vision-based DSS for crack detection”. They offer first insights into a nascent design theory for the application case of road crack detection on the basis of images.

Not only roads, but also railways need to be inspected periodically to ensure safe transports. Wei et al. (2019) employ a Faster R-CNN to detect defects of railway track fasteners. Gibert et al. (2017) propose a CNN-based multitask learning approach that detects railway track fasteners and crossties and classifies the state of these components.

Wind turbine blades are another physical object of interest for CV-based infrastructure inspection. Akhloufi and Benmesbah (2014) present a CV approach to identify ice accreation on wind turbine blades. Ice accreation can require a maintenance action since it can cause malfunction and premature wear and is a safety hazard for nearby people and infrastructure like roads and powerlines. Shihavuddin et al. (2019) show how faults like leading edge erosion can be detected with a Faster R-CNN on wind turbine blades.

### Automated UAV Vision-Based Approaches for Power Line Inspection

In this work, we are particularly interested in CV solutions for power line inspection relying on UAV images. From a component-based view, power line inspection can be divided into four major categories: towers or poles, insulators, conductors, and fittings (Liu et al. 2020). Each of these categories contains several subcomponents (Nguyen et al. 2018) that typically vary in size, kind, and material according to the voltage level. For instance, some part of a distribution network with low voltage might have wooden poles, small standing insulators, and a single, relatively thin conductor. On the other hand, transmission networks usually have lattice steel towers, large suspending insulators, and thicker conductors. Several studies have been published that utilize various potential platforms (e.g., helicopter, satellite, and UAV) to collect different data types (e.g., optical images, laser scanner data, thermal images, and synthetic aperture radar images) and analyzed these through different processing techniques (Matikainen et al. 2016). The vision-based approach – with image data from the visible spectrum captured by UAVs and automatically analyzed through image processing capabilities – has gained the most attention and traction in the power line inspection research domain (Liu et al. 2020).

With a few exceptions, automated vision-based power line inspection based on UAV-captured images requires two inherently related tasks (Liu et al. 2020): component detection and localization as well as fault diagnosis. The exceptions relate to objects such as bird nests, whose detection already represents a fault. Previous research applying image processing for the detection and fault diagnosis of PLCs is numerous (Mirallès et al. 2014). Liu et al. (2020) identify several characteristics and shortcomings of previous studies using UAV-captured images in their exhaustive literature review. Most studies in the field of vision-based inspection of power lines focus on the insulator and its faults (Liu et al. 2020) – mainly missing caps (e.g., Sampedro Pérez et al. 2019; Yang et al. 2019; Zhai et al. 2018) – while little attention has been paid to other components. The safety pin that prevents other components from loosening and falling is the smallest object in the power line and has, despite its importance, received little attention and has only been regarded in fault diagnosis but not in the detection step. Finally, both Liu et al. (2020) and Nguyen et al. (2018) conclude that the mediocre performance of task-specific approaches presented in the vast majority of studies has been superseded by DL approaches that have improved the performance of component detection as well as fault diagnosis.

To move towards the operationalization of automated vision-based inspection, we require approaches capable of detecting a wide variety of components and diagnosing their faults in order to integrate them into a valuable system. Although “the component detection is a relatively mature area” (Liu et al. 2020)[p. 10], we found that only a few articles shed light on detecting several components in a single approach or pipeline. Besides the identified challenges, we therefore review all available DL-based approaches that consider more than one component in the detection step.

The first steps in this field were done by Zhu et al. (2018), who investigate the cascading of two Faster R-CNN architectures for high-voltage PLCs. While towers, spacers, vibration dampers, and insulators are directly detected from the input image on the first stage, the pixel coordinates of the tower are used to crop the input image and consequently feed it into the second stage to detect small objects – in their case bird nests and tower plates. Their results show that the cascaded architecture is able to detect small objects at better performance. Nguyen et al. (2019) propose a similar approach for low-voltage PLCs (pole, cross-arm, insulator, or top cap) with a large number of various subcomponents totaling 54 classes. The authors detect poles in the first stage, crop the respective image and detect other, smaller components in the second stage. In a third stage, the recropped components are fed into image classifiers to perform a fault diagnosis. This work shows the feasibility of designing a cascaded multistage detection and classification pipeline utilizing spatial relationships. However, it does so only for larger components in terms of pixel size. Liang et al. (2020) take a different approach. They do not follow the prevalent approach of separating detection and fault diagnosis, but skip the general detection of PLCs and directly detect only components that exhibit faults. While including a total number of ten fault types, the work naturally states the problem of the detection of intact components as defective components. It also does not try to achieve the detection or fault diagnosis of overly small components.

The aforementioned approaches can strongly facilitate inspection and thus the prioritization of subsequent maintenance operations. Additionally, the data that is acquired in an automatic and structured manner can serve as foundation for predictive maintenance (Selcuk 2017). By utilizing the data to train detection models (as shown later in this work), continuous forecasts about the future occurrence of defects can be issued. A well-trained and deployed model can, therefore, support experts in indicating future maintenance needs early and prioritize potential work orders.

### Image-Based Decision Support Systems

The access to increasing volumes of images and the capabilities of DL to process and extract information from images creates the potential to harness this rich data and DL methods to facilitate effective decision-making (Chaudhuri and Bose 2020). Despite their capabilities, DL methods, particularly CNNs, have found limited adoption in extant research of IS in general (Kraus et al. 2020), and specifically DSS. Most research performed on image-based decision support focuses on the medical application domain (Ben-Cohen et al. 2017; Comaniciu et al. 1999). However, these works use highly specific medical scans rather than images from the visible spectrum. Some examples of the scarce literature on DL-enabled image-based decision support in non-medical contexts include vision-based maintenance and monitoring applications or pattern analysis (Xie et al. 2020; Schumann et al. 2019; Chaudhuri and Bose 2020; Nazerdeylami et al. 2019; Jamshidi et al. 2018; Ren et al. 2020).

Despite the efficacy of DL methods for image processing in related decision support contexts, none of the previous work provides guidance on how to design IB-DSS. Specifically, although all these studies aim for improved data and information availability, close to no insight is provided on how to bridge the gap between the sole image processing as well as consequent information extraction, and the respective efficient, high-quality decision-making.

### Synthesis and Research Gap

This work aims to interweave two research domains. It combines the applied research of image processing in power line maintenance (PLM) with the need for decision support in vision-based domains in general and in PLM in particular. This allows us to tap new potential through making previously unattainable data and information from individual images available.

We address this potential by investigating the environment of automated vision-based PLC maintenance, focusing on the design of a holistic image-based decision support solution. We develop design knowledge for IB-DSS and evaluate it by instantiating a concrete artifact for PLC maintenance. We extend the reviewed existing works (cf. Table 1) by managing to detect PLCs of extreme size difference (insulators and safety pins), which we believe is a crucial prerequisite for moving towards decision support in this domain.

## Research Methodology

The research at hand develops design knowledge for IB-DSS which supports the maintenance decision-making and planning of maintenance engineers (MEs) for power lines. Since design science research (DSR) has proven itself to be not only a suitable but also an important paradigm to develop IS in general (Gregor and Hevner 2013) and DSS in particular (Arnott and Pervan 2012), we follow its steps to develop and evaluate our artifact. At its core, DSR is a problem solving paradigm that involves two primary and distinct activities to design solutions to real-world problems: (1) the development of innovative artifacts in a series of design activities based on a deep understanding of the problem, justificatory knowledge, and the capabilities of the researcher and (2) the evaluation of the novel artifact to assess its ability and utility in solving the identified problem (Hevner et al. 2004). Following this “build-and-evaluate loop” (Hevner et al. 2004), we iteratively develop an artifact to extend the knowledge base regarding IB-DSS.

Besides this loop – more precisely termed design cycle – Hevner (2007) describes the existence of two additional cycles: relevance and rigor. The three cycles are inherently related and part of any DSR project. The relevance cycle connects the environment, application domain, or case company of the research project to the design science activities by, for instance, incorporating input from expert practitioners. It does not only provide the requirements, problems, or challenges for the research, but also defines acceptance criteria (Hevner and Chatterjee 2010). The rigor cycle relates the design science activities to the existing knowledge base. It provides knowledge from scientific theories, engineering methods, experience, and expertise to the research project. The often repeatedly performed design cycle is the core of any DSR project and naturally builds on the insights from the two previous cycles. Specifically, during a design cycle the research iterates between construction and evaluation of an evolving artifact (Hevner and Chatterjee 2010) to eventually deploy the artifact in the environment as well as distill insights and output the research’s design knowledge contributions into the knowledge base.

**Fig. 1:**
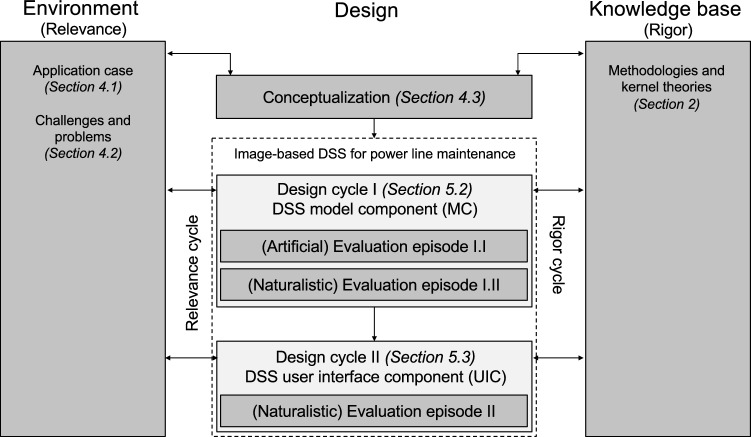
Overview of the research cycles and activities in the conducted study (based on Hevner 2007)

In the general view of our research displayed in Fig. 1 we start with studying the environment in which the research is embedded. We consequently state our application case (Sect. 4.1) and review related challenges and problems (Sect. 4.2). Joining these insights with knowledge from kernel theories we conceptualize principles and requirements for the problem class of IB-DSS. We subsequently derive a concrete PLM artifact and, based on Turban et al. ’s (2010) high-level notion of a DSS, first focus on the *model component* (MC) of our DSS artifact in the first design cycle (Sect. 5.2). Afterwards we move to the *user interface component* (UIC) in the second design cycle (Sect. 5.3). To orchestrate the evaluation of our artifact, we apply and follow the overarching *Framework for evaluation in design science* (Venable et al. 2016) to rigorously demonstrate the utility and efficiency of the artifact and its underlying design knowledge. Figure 1 provides an overview of the performed evaluation episodes (EE) in these design cycles. As it is our goal to indicate technical feasibility as well as utility of IB-DSS enabled through DL, we start with a technical evaluation and then move to a naturalistic context within the application setting.

## Application Case and Conceptualization

Our DSS artifact, built on images from the visible spectrum, intends to support MEs of power line infrastructure in their decision-making. More precisely, our system supports the planning and scoping of individual maintenance orders for the repair and replacement of components through improved data and information quality. Because the artifact is to intervene in an organizational context, it is considered “socio-technical” (Gregor and Hevner 2013). To manage the complexity of the artifact construction in terms of size as well as social and technical components, Gregor and Hevner (2013) suggest the explicit extraction of design principles (DPs). We therefore conceptualize and suggest a number of tentative DPs for the design of artifacts of the problem class of IB-DSS by first investigating challenges in power line maintenance (PLM). These are recast into a prescriptive mode with appropriate abstraction yielding preliminary design requirements (DRs), which then serve as a basis for deriving the DPs.

### Application Case and Decision Process

As the largest distribution system operator in Baden-Württemberg, Netze BW supplies around 2.2 million customers and operates a network of almost 100,000 km. The distribution network, which is largely rural, poses challenges in the inspection of towers, poles, and overhead line routes. Every year, Netze BW operators routinely carry out around 7000 scheduled inspections of high-voltage towers and lines, which include a visual inspection from the ground or by helicopter. For around 1400 of these, towers must be climbed physically. Whenever operators identify an issue or defect on a tower during these inspections they manually create a report including the location, description, and if possible images. Based on these largely unstructured inspection reports, MEs need to subsequently compile situation-dependent, well-defined, complete, and prioritized maintenance orders. Accordingly, based on reported incidents MEs first scan the report and verify the priority of the incident. While the priority determines the processing order, for any incident several maintenance order specific details need to be compiled regardlessly. MEs will therefore check the topology surrounding an incident location as it determines which device and equipment can be used. Additionally, the incident and its preferred solution approach determine whether either internal operators can be dispatched or contractors are required. Another important aspect especially for incidents of lower priority is the consideration of forthcoming infrastructure revision projects. These can typically include the required maintenance order and, thus, avoid additional work. Finally, to avoid hazards during the maintenance work the respective circuits must be free of electrical current which requires routing the current flow to other power lines. Hence, MEs need to appropriately terminate these so called switches based on the incident priority and in close consultation with the grid control center as well as operators or contractors. Since the electrical grid often offers small margins for additional current flow such switches are often times difficult to set up. On this occasion MEs need to bundle incidents on the same power line to use such switches as efficiently as possible.

### Challenges in the Power Line Maintenance

To understand PLM from a practitioner as well as a theoretical perspective, we started our research with a series of expert interviews among the case company’s employees and a structured literature review (SLR) of domain-specific articles. The interviewees were chosen based on their their work experience and affiliation to different departments dealing with the various aspects of the PLM process (cf. Table 2). This sampling allowed us to benefit from diverse viewpoints and nuanced perspectives on the challenges of PLM with today’s manual inspection.

**Table 2:** Overview of interview participant to determine challenges in power line maintenance

ID	Role	Experience [Years]
Alpha	Senior standardization engineer	10
Beta	Operator of high- and medium-voltage power lines	25
Gamma	Operations manager of high-voltage power lines	28
Delta	Asset manager	12

To guarantee a rigorous overview, we conducted the SLR following Webster and Watson (2002) and vom Brocke et al. (2009) by querying various databases (cf. Table 3). We harnessed a selection of search strings, as displayed in Table 3, to retrieve the initial set of relevant articles. To extract only relevant articles, we defined three exclusion criteria. If the paper examined or investigated only one specific solution approach for the automation of PLM, it was excluded. If a paper focused on constant monitoring of power lines rather than periodic inspection, it was also excluded. Finally, if on a thorough read of the paper no challenges regarding PLM were mentioned, the paper was ruled out. These exclusions allowed us to focus on review and survey contributions for the automation of PLM. The SLR conducted in January 2020 resulted in a large number of potentially relevant contributions as depicted in Table 3, with 22 papers remaining after the first exclusion and 18 survey and review papers mentioning challenges in today’s PLM.

**Table 3:** Search strings and respective results for the structured literature review

Search strings	EBSCO	WoS	IEEE Xplore	Scopus
“Automat*” AND “Power line” AND “Inspection”	24	79	86	158
“Power line” AND “Quality control”	4	89	12	141
“Transmission line” AND “Automat*” AND (“Inspection” OR “Monitoring”)	21	97	213	370
“Inspection” AND (“Power line” OR “Transmission line”)	104	393	547	1301
(“Power line” OR “Transmission line” OR “Overhead lines” OR “Overhead power lines”) AND “Condition monitoring”	18	131	0	271
“Challenges” AND “Power line” AND “Inspection”	2	7	8	24

Statements from both the interview transcripts and scientific articles were then coded in an open coding process and combined in a qualitative content analysis as proposed by Mayring (1991) to derive a category system of today’s PLM challenges. Table 4 on page 14 depicts part of the identified challenges with the respective subchallenges and their sources. These three challenges (C1–3) appeared to be specific to our context of infrastructure inspection with its concrete characteristics being dependent on power line infrastructure and therefore inform the design of our artifact. Further identified challenges attributed to company and industry specifics can be found in Sect. A1 within the Appendix (available online Supplementary material).

**Table 4:** Challenges in the maintenance of power lines based on expert interviews and a structured literature review

ID	Challenge	Subchallenge	Source
C1	Complicating workplace characteristics	C1.1–Hazardous work environment	Pagnano et al. (2013), Nguyen et al. (2018), Jones (2005), Li and Wang (2019), Seok and Kim (2016), Huang et al. (2018), Toth and Gilpin-Jackson (2010); Alpha; Beta
		C1.2–Strenuous inspection activities	Alpha
		C1.3–Requirement for broad expertise	Takaya et al. (2019), Pernebayeva and James (2020), Huang et al. (2018); Alpha; Beta; Gamma; Delta
		C1.4–Impact of subjectivity	Nguyen et al. (2018), Jones (2005), Katrasnik et al. (2010), Toth and Gilpin-Jackson (2010), Homma et al. (2017); Beta; Delta
C2	Inspectability challenges	C2.1–Inspection type related scope restrictions	Jones (2005), Katrasnik et al. (2010);Beta; Gamma; Delta
		C2.2–Requirement for unscheduled inspections	Matikainen et al. (2016)
C3	Infrastructure characteristics	C3.1–Age of power line infrastructure	Aggarwal et al. (2000), Toussaint et al. (2009); Alpha
		C3.2–Extent of power line infrastructure	Pagnano et al. (2013), Aggarwal et al. (2000), Pernebayeva and James (2020), Huang et al. (2018), Homma et al. (2017); Alpha
		C3.3–Topography of infrastructure territory	Prasad et al. (2016), Deng et al. (2014), Aggarwal et al. (2000), Takaya et al. (2019), Pernebayeva and James (2020), Matikainen et al. (2016), Seok and Kim (2016), Huang et al. (2018), Toth and Gilpin-Jackson (2010), Homma et al. (2017)
		C3.4–Vast spectrum of inspection aspects	Nguyen et al. (2018), Prasad et al. (2016), Jones (2005), Homma et al. (2017); Alpha; Gamma

### Design Requirements

**Fig. 2:**
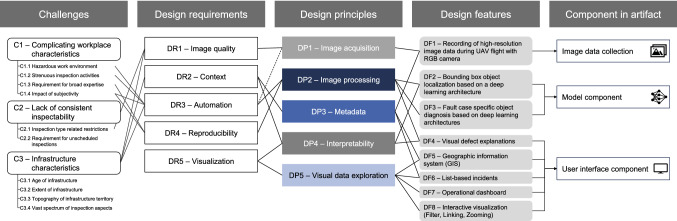
Design knowledge for image-based decision support systems with its respective instantiations

Our DSS artifact intends to support MEs of power line infrastructure in their planning and scoping of individual maintenance orders to repair and replace components. To accomplish this by systematically addressing the aforementioned uncovered challenges in PLM with a vision-based application, we cast these challenges into a prescriptive mode and derive DRs as depicted in Fig. 2. Consequently, we derive five DRs which describe our system objectives and confine to which objectives our subsequently derived design knowledge applies (Walls et al. 1992). Because we target developing generalized design knowledge for the problem class of IB-DSS, we formulate the DRs on the relevant level of abstraction in the following.

The infrastructure characteristics (C3.1–C3.4) pose challenges with regard to efficient data capturing as, for instance, power lines running across valleys or in mountainous areas complicate inspection and hinder data acquisition. In addition to this, the three inspection types used in today’s PLM provide heterogeneous condition data of varying quality (C2.1). Together, these factors result in the need for an appropriate *image quality* relating to uniformly captured high-resolution image condition data regardless of infrastructure characteristics and with process consistency.

*DR1 – Image quality:* The system should uniformly capture condition image data of sufficient quality.

Image data contains large amounts of unstructured information. However, the information contained in an image is typically of little use if its observer lacks contextual information. Context allows for a broader understanding of specific pieces of information and it places them in a bigger picture by for example providing temporal or geographical information. Images of the infrastructure and in particular of components therefore need to be contextualized in an appropriate way. On the other hand, the infrastructure characteristics (C3.1–C3.4) pose the requirement for providing infrastructural context to enhance decision-making.

*DR2 – Context:* The system should capture and provide context.

Today’s inspection process of power line infrastructure is fully manual and labor-intensive. Above that, various human-factor-related challenges (C1.1, C1.2, and C1.4) influence the inspection’s susceptibility to errors. Additionally, characteristics of the infrastructure, such as topography (C3.2) and extent (C3.3), result in an increased labor effort for maintenance. To mitigate the limitations of today’s inspection process, both parts of the process – image acquisition and image processing – should be infused with *automation* capabilities.

*DR3 – Automation:* The system should allow for automatic image acquisition and provide automated image processing.

To make adequate maintenance decisions in terms of repair or replacement prioritization, MEs require consistent condition data. However, just as in any human-based inspection, the fault diagnosis of power lines is characterized by the personal experience and expertise (C1.3) of the inspector, making the evaluation or judgment subjective (C1.4). To objectify the fault diagnosis and making it less subject to the experiences of a wide variety of inspectors, personal biases need to be eliminated or harmonized. Consequently, the analysis of the condition data needs to build upon equal decision parameters, achieving reproducible results. By *reproducibility* of results we refer to similar evaluation or fault diagnosis of a unique PLC within a range of potentially changing environmental conditions (e.g., lighting conditions).

*DR4 – Reproducibility:* The system should provide image processing in a reproducible manner.

To draw inferences from the previously captured data and extract crucial information, proper visualization is required. Consequently, not only the quality but also the presentation of information regarding faults in the power line infrastructure are crucial. Specifically, it is important to integrate and transfer the entire collected data from the data acquisition and the extracted information from the data processing into the maintenance decision-making to enable the compilation of situation-dependent, well-defined, complete, and prioritized maintenance orders.

*DR5 – Visualization:* The system should support the process of decision-making with the visualization of the extracted information.

### Design Principles

In the following, we suggest several design principles (DPs) which prescribe how to develop the artifact in order to accomplish our predefined preliminary DRs (Chandra et al. 2015). The translation process from DRs into tentative DPs is displayed in Fig. 2. The DPs use the knowledge of several theories in order to meet the DRs. The main contributions originate from the domains of image processing, DL, DSS, as well as visual data exploration.

We have identified that the images of the PLCs need to be captured uniformly and with sufficient quality (DR1). Additionally, the system should capture context (DR2) of the images for unambiguity regarding their location and time. To address these design requirements, two considerations have to be made: the type and kind of data collected and the collection method, which we will refer to as platform. The primary type of collected data is predefined in our use case to be image data from the visual domain as it (1) provides enough information to detect a wide variety of common faults (Nguyen et al. 2018) – especially on PLCs – and (2) allows fast comprehension by MEs. On the other hand, the platform responsible for the data acquisition needs to be able to acquire uniform image data. In particular, the platform should be able to combine the advantages of today’s inspection methods of helicopter-based, ground-based, and climbing-based inspection in a way that each of these methods that are specifically suitable for different components can be imitated. The platform is consequently able to capture images from above, below, and the front while maintaining a uniform viewing perspective per component type. The system should also allow data acquisition to happen in a potentially automated fashion (DR3) to further increase the scalability and reduce human involvement in the inspection process.

*DP1 – Image acquisition:* Provide the system with (automated) capabilities for uniform acquisition of images in context.

The system relies on a vision-based approach with captured images containing information about the infrastructure condition. The image data should be processed in an automated and reproducible fashion (DR3 and DR4). Image processing is necessary to process and analyze the data in order to extract the desired information. Image processing has traditionally been implemented for industrial applications like quality control of manufactured parts, as they exhibit inherently less challenging lighting conditions and scene complexity than outdoor environments (Mirallès et al. 2014). Owing to the rapid growth and evolution of DL (Liu et al. 2020; LeCun et al. 2015) in general and CNNs in particular, there are many successful approaches that have improved the performance of visual recognition systems in application areas such as self-driving cars, face recognition, image search, and image understanding (Nguyen et al. 2018) despite the challenging conditions of outdoor application. CNNs provide a method for automatically learning features in images, which can drastically reduce the effort in hand-designing solutions and improve generalization. In summary, this makes its application promising for the analysis of images containing PLCs (Jalil et al. 2019; Sampedro Pérez et al. 2019; Prates et al. 2019). Consequently, based on the assumption that all relevant components are captured in images, they can be extracted using DL. In particular, the assessment of a component’s condition features is determined by two factors. First, the component needs to be detected in the captured image, containing one or more component objects. Second, each detected component requires component-specific fault diagnosis. The system should therefore include these two tasks performed by a DL approach.

*DP2 – Image processing:* Provide the system with state-of-the art deep learning for the detection and fault diagnosis of components.

Images containing PLCs form the basis of the IB-DSS for vision-based maintenance. However, without any additional information the images can hardly be seen as sufficient for a system designed for component maintenance. To enable MEs in their decision-making, *metadata* (Sen 2004) regarding the images or contained components is required. The primary purpose of this metadata is to provide context (DR2) to the reported data and therefore provide enriching information that leads to knowledge creation (Nicola 2005). It can describe both physical (e.g., towers and insulators) as well as digital objects (e.g., images and documents) through providing values or information for certain characteristics (Clobridge 2010). The main purpose of attaching metadata to a data item is to uniquely identify it in a system and to find it by browsing or searching (Burgin 2016). In the PLM, metadata can range from geographical and temporal image tags all the way to geographical location, age, history, et cetera of the individual infrastructure components. However, the main consideration to be taken here is that the physical objects, such as towers, insulators, or conductors, are to be considered the focal data as they represent the maintained infrastructure. The captured images contain information about these components and should therefore be appropriately linked, at best based on the individual component.

*DP3 – Metadata:* Provide the system with metadata.

The availability of context in the form of simple metadata such as the geographic location and a time stamp or advanced/processed metadata such as the object location, object type, and binary fault presence adds valuable information to an IB-DSS. However, in terms of context for the individual fault contained in an image, these details are of limited help. In the light of fault diagnosis, the required context (DR2) should be defined as parts of it that can be accessed to clarify and understand the fault. The combination of the contextualized fault diagnosis as well as visualization of the extracted information (DR5) directly results in necessary interpretability of the decision in the fault diagnosis. Consequently, the decision of the fault diagnosis should be interpretable for MEs such that they are able to comprehend why for instance an insulator was marked as faulty. Thereby, we adapt the definition of Miller (2019)[p. 14] referring to interpretability as “the degree to which an observer can understand the cause of a decision”. The interpretability of the results of the fault diagnosis provides MEs with additional information (context) at a PLC level which in turn enhances their ability to make high-quality decisions.

*DP4 – Interpretability:* Provide the system with interpretable fault diagnosis.

To facilitate decision-making in PLM, we found that acquired and processed data should be visualized (DR5) to the respective users in order to determine a fault’s existence, location, and significance. Because such a user interface can be considered as the ”source of many of the power, flexibility, and ease of use” (Turban et al. 2010, p. 100) of a DSS, it requires careful consideration. MEs face a situation where they need to compile well-defined, complete, and prioritized maintenance orders with a variety of details and latent information requiring their consideration. An appropriate interface should therefore harness visual data exploration (Keim 2002) by integrating its user into the data exploration process by applying their perceptual abilities. It can help the personnel to answer the mission critical questions such as the required equipment and achieve high decision quality regarding maintenance prioritization.

*DP5 – Visual data exploration:* Provide the system with an interface for visual data exploration.

## Image-Based Decision Support System for Vision-Based Power Line Maintenance

To improve the planning and scoping of individual maintenance orders, enhanced data and information quality needs to be provided to MEs. By following the prescribed tentative DPs for an IB-DSS our designed and evaluated artifact provides evidence of achieving this objective. The artifact is integrated into our case company by deriving specific capabilities to satisfy the DPs, termed design features (DFs) (Meth et al. 2015). Accordingly, we present the image data collection, their subsequent processing and analysis through the MC, and the presentation of the results through the UIC along with their respective DFs depicted in Fig. 2 (cf. page 15) in the following three subsections.

### Image Data Collection

The platform responsible for the image data collection is required to capture images of sufficient quality. Consequently, it needs to be able to acquire uniform, standardized, and consistent image data in a potentially automated way (DP1). UAVs equipped with capabilities to capture optical images (DF1) meet these expectations (Nguyen et al. 2018; Matikainen et al. 2016; Spencer et al. 2019) for our specific use case. This is due to three main reasons. First, UAVs are able to capture images from above, below, and the front, combining the best aspects of today’s helicopter, ground, and climbing inspection methods. Second, a UAV’s ability to fly close to power lines allows it to take detailed images. Finally, although an approach for UAVs’ autonomous navigation and image acquisition along power lines still has to be developed, the general feasibility of this automation step is undisputed (Nguyen et al. 2018).

### Deep-Learning-Enabled Model Component

To build an efficient IB-DSS for infrastructure maintenance, images containing relevant components, meaning components that exhibit faults, need to be identified from the entire dataset. For this purpose, we present the preparation, instantiation, and evaluation of our MC below.

#### Data Description and Preparation

To build a DL vision-based MC, large quantities of data are required. We therefore collected images of PLCs, annotated them according to our desired component classes, prepared them for training through creation of several datasets, and finally used them for model training.

**Fig. 3:**
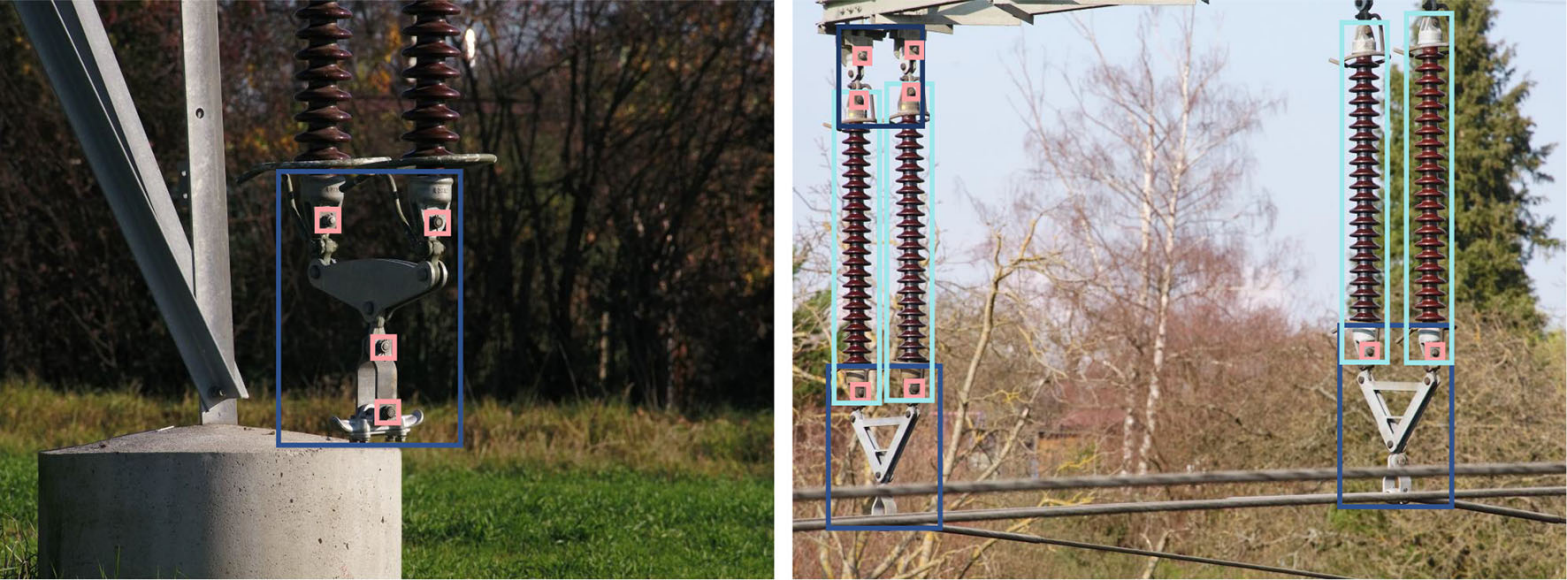
Exemplary images of the $DS_{Ro}$ dataset containing *insulators* (cyan), *fittings* (blue and dark blue), *birdnest* (not present), and *safetypins* (pink). The images show various subcomponents of the component types, captured from varying perspectives to ensure the robustness of the model; the left image provides an impression of the artificial setup for capturing defective *safetypins* (color figure online)

The images were collected by flying a UAV along high-voltage power lines in several selected areas in southern Germany and circling around power towers to take pictures of PLCs. The power line passages were selected so that the captured images would contain diverse background scenes and PLCs of varying age and type. For each power tower, around 70 images were captured. Images containing faulty safety pins were created artificially in collaboration with field experts. Accordingly, an insulator and fitting application was installed in the lower area of one power tower (see Fig. 3 – left image) and a sequence of 608 images was captured while modifying the splint itself as well as changing the respective image perspective.

**Table 5:** Characteristics of the datasets

Dataset	# Images	Image resolution	Volume	Annotation type	# Annotation	Objective
$DS_{Ro}$	1690	5280 × 3956	15.2 GB	BB + label	9182	Single-stage component detection (*insulator*, $fitting_{top},$ $fitting_{bottom},$ *birdnest*, *safetypin*); derive data set $DS1_{Co}$, $DS2_{Fi}$, and $DS3_{Pi}$
$DS1_{Co}$	1589	5280 × 3956	14.3 GB	BB + label	3996	Multistage large component (*insulator*, $fitting_{top},$ $fitting_{bottom},$ *birdnest*) detection
$DS2_{Fi}$	1820	1200 × 1200	1.2 GB	BB + label	5186	Multistage small component (*safetypin*) detection from cropped $fitting_{top}$ and $fitting_{bottom}$
$DS3_{Pi}$	5186	60 × 60	35.3 MB	Label	5186	*safetypin* fault diagnosis

**Fig. 4:**
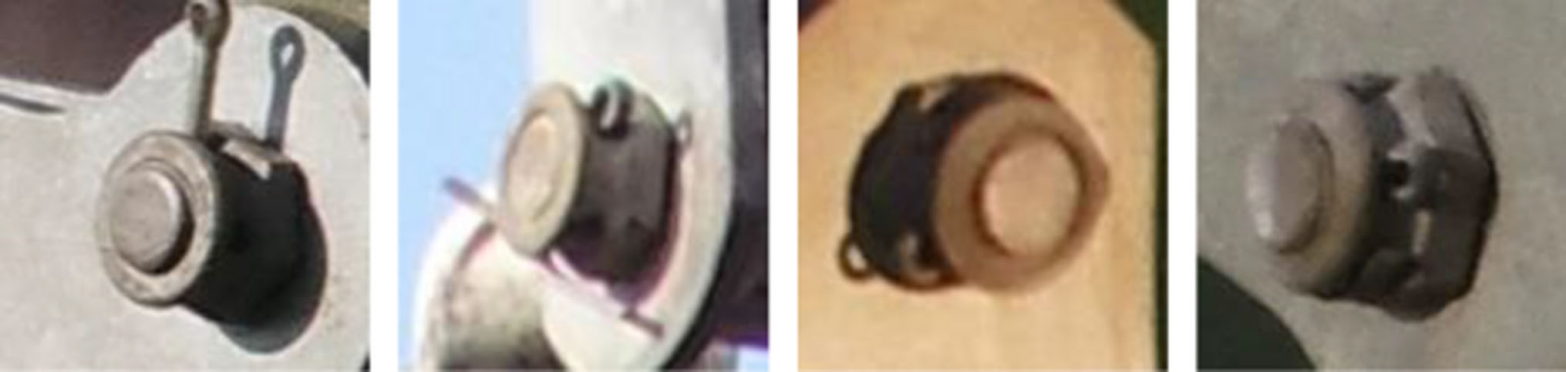
Exemplary images of the *safetypin* component type from the $DS3_{Pi}$ dataset. The defective *safetypins* (two to the left) are not completely bent, while the intact ones (two to the right) are completely bent and consequently prevent slipping out

After collecting the images, each one was annotated with bounding boxes ($BB_{GT}$) representing the ground truth. Each *BB* was associated with one of five PLC classes (*insulator*,  $fitting_{top},$ $fitting_{bottom},$ *birdnest*,  *safetypin*) that we chose for this project. These annotations and the respective images eventually constituted our root dataset $DS_{Ro}$, containing 1424 *insulators*, 1073 $fittings_{top}$, 1438 $fittings_{bottom}$, 61 *birdnests*, and 5186 *safetypins*. Two further datasets $DS1_{Co}$ and $DS2_{Fi}$ were obtained through subsampling $DS_{Ro}$ to train different aspects of the object detection as depicted in Table 5. Finally, $DS3_{Pi}$ was derived to train the classifier for *safetypins*, with 1494 images of defective and 3692 images of intact *safetypins*. The characteristics of the four datasets are summarized in Table 5 and sample images are shown in Figs. 3 and 4 (cf. page 21).

#### Instantiation of a Multistage Pipeline

**Fig. 5:**
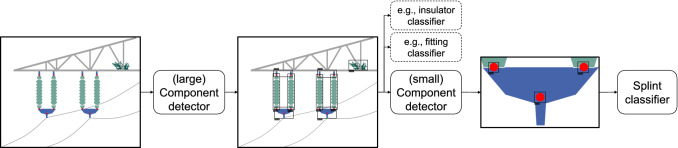
Structure of our multistage power line component detection and classification pipeline for high-resolution images

Inspired by Nguyen et al. (2019) and Liu et al. (2020), we designed a DL-based multistage component detection (MSCD) and classification pipeline for high-resolution images containing multisized objects with spatial relationships (DF2 and DF3) to satisfy DP2. This addresses the requirement for automation (DR3) of infrastructure inspection (Katrasnik et al. 2010; Montambault et al. 2010 and reproducability (DR4) of the derived results to mitigate subjective decisions (Katrasnik et al. 2010; Toth and Gilpin-Jackson 2010). While our case company is interested in the fault diagnosis of a significantly larger number of components, for the purpose of this study we intend to only demonstrate the feasibility of detecting both the smallest components (*safetypins*), as well as the largest ones (*insulators*), in images taken of high-voltage power lines – a topic not yet considered in the automated inspection of power lines. The pipeline consists of three elements responsible for different detection and classification tasks, as displayed in Fig. 5.

In the proposed MSCD pipeline, the *(large) component detector* first detects *insulator*,  $fitting_{top},$ $fitting_{bottom},$ *birdnest* from an input image. The detected *fittings* are cropped from the input image and used as input for the subsequent *(small) component detector* to detect *safetypins*. The detected *safetypins* are recropped and passed into the *pin classifier* for fault diagnosis.

For the implementation of the MSCD, we chose to compare two well-proven DL object detection architectures – SSD (Liu et al. 2016) and Faster R-CNN (Ren et al. 2015) – which we additionally benchmarked against a single-stage component detection pipeline (SSCD), meaning all components are detected in one step. We selected ResNet as the backbone CNN for the object detection architectures as well as our main classifier for the fault diagnosis of the *safetypins*. To compare and benchmark the fault diagnosis, we chose the well-known VGG16 (Simonyan and Zisserman 2015) architecture. In both tasks, image augmentation was used to improve the generalization of the models. For object detection the brightness of the images was randomly adjusted. For the classification task, where cropped images of *safetypins* were classified, we applied horizontal and vertical flipping, random brightness adjustment, width as well as height range shifting, and random image blurring.

The component detectors were implemented using the Tensorflow^3^ DL framework^4^ (Abadi et al. 2015) with models pretrained on the MS COCO dataset (Lin et al. 2014). The image classifiers were realized using the Keras DL library^5^ (Chollet et al. 2015) which provides image classification models pretrained on the ILSVRC dataset (Russakovsky et al. 2015).

#### Evaluation of the Instantiated Model Component (EE I.I & EE I.II)

For the evaluation of DF1–DF3 and DP1 and DP2 respectively, we conducted both an artificial evaluation to closely assess the pipeline’s efficacy and efficiency as well as a naturalistic evaluation to generally judge the design’s acceptance and usefulness. In accordance, the evaluation episodes were guided by the questions below:

- **EE I.I ** How well does the proposed DL-based MC extract power line components of various sizes? How well does it diagnose component faults?
- **EE I.II** Do MEs regard the MC’s capabilities as helpful?

**Artificial evaluation of the model component (EE I.I)**

The efficiency evaluation of the proposed pipelines required two considerations. First, the pipeline’s ability to detect the chosen components needed to be evaluated. Second, the accuracy of the fault diagnosis – which we performed for detected *safetypins* – had to be assessed.

**Fig. 6:**
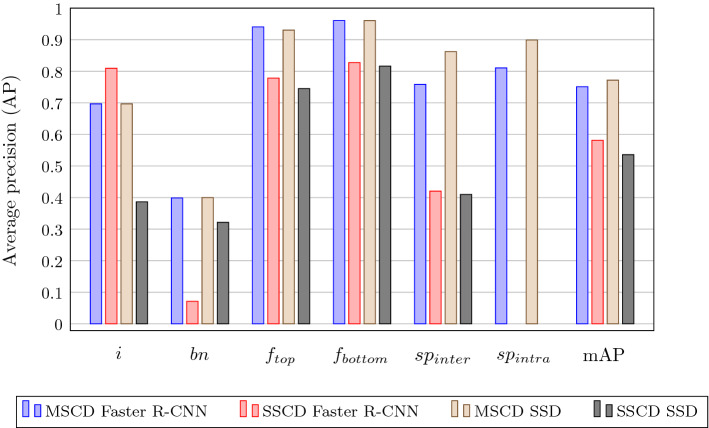
Average precision of the different pipelines using Faster R-CNN and SSD on the five selected component classes

Evaluating the efficacy and efficiency of the detection task in terms of average precision (*AP*) and mean average precision (*mAP*) (Rafael Padilla and da Silva 2020), we compared our proposed MSCD to the SSCD pipeline. As we were working with our own proprietary dataset $DS_{Ro}$, the available images were split into a training set comprising 80% of the data, with the remaining 20% used for the evaluation set. To increase the evaluation’s validity, images captured at one tower were held out from the random split and solely utilized for the evaluation dataset, while maintaining the split ratio. This image-level split was kept consistent across the derived datasets $DS1_{Co}$ and $DS2_{Fi}$. The SSCD pipeline was fine-tuned to detect the respective component classes using the $DS_{Ro}$ dataset. Accordingly, both detection stages of the MSCD pipeline were fine-tuned on $DS1_{Co}$ and $DS2_{Fi}$ respectively. All models were trained using the stochastic gradient descent optimizer with 0.0003 (Faster R-CNN) and 0.001 (SSD) initial learning rate respectively, 0.9 momentum, and batch size 64. We determined the models by using early stopping on the validation loss with a patience of 100 for all models. The testing results of the different pipelines using the different architectures are shown in Fig. 6. The performance for the *safetypin* class is disclosed in terms of *inter* pipeline performance for both the SSCD and the MSCD pipeline as well as the *intra* pipeline performance for solely the MSCD pipeline.

We evaluated the fault diagnosis task performed for the *safetypins* class in terms of weighted precision, weighted recall, and weighted $F_1$-score (Pedregosa et al. 2011) to account for class imbalance. We applied a 3-fold cross validated grid-search to identify the optimal combination of parameters. We chose to account for the following parameter: unfrozen convolutional layers, dense layer size, optimizer and its respective learning rate, dropout rate, and batch size. The images in dataset $DS3_{Pi}$ were shuffled, a hold out set containing 10% of the images was retained and the remaining images were split into 3 folds. Consequently, for each grid search configuration three models were trained with early stopping with patience 30. The best resulting model of the Resnet and VGG16 model were harnessed to be evaluated on the retained hold out set. The results of the evaluation of the cropped *safetypin* classification task based on the test set are shown in Table 6. All details on the machine learning steps and choices are depicted within Sect. A2 in the Appendix (Kühl et al. 2021).

**Table 6:** *Safetypin* crop classifier test results on the $DS3_{Pi}$ dataset

Architecture	AUROC	Weighted precision	Weighted recall	Weighted F1-score
VGG16	0.8114	0.80	0.80	0.78
ResNet50	0.8080	0.76	0.75	0.71

**Naturalistic evaluation of the model component (EE I.II)**

To answer whether the detection and fault diagnosis of PLCs help MEs, we conducted nine purposefully sampled (Coyne 1997) interviews with potential users of the IB-DSS from our case company. The interviewees included two senior MEs (Epsilon – Zeta) with a working experience of 34 and 41 years, five MEs (Eta – Lambda) with on average 27 years experience, one operations manager (My) with 28 years’ working experience, and one senior standardization engineer (Ny) with 10 years’ working experience. Each interviewee received a brief introduction to the DF1-DF3. Accordingly, the image data collection setup employing UAVs and the image analysis to detect and diagnose PLCs was introduced. Exemplary images (cf. Fig. 3, page 21) were shown to clarify the use case. The interviewees were allowed to ask questions of comprehension. Subsequently, in a semi-structured interview fashion each participant was asked to evaluate the DFs. A detailed overview of the questionnaire can be found in Appendix A3 on page 40. The question of whether each presented DF appropriately addresses its respective DPs served as the starting point. The interviewees opinion and attitude regarding all DFs was explored and probing questions were asked if necessary. This allowed us to assess the attitude of human expert workers towards the technology. This initial evaluation of part of the IB-DSS’s tentative design serve as initial mediation to ensure that the final artifact can be designed as a useful and efficient instrument for solving our research question.

In accordance with Hevner and Chatterjee ’s (2010) suggestion for the analysis of confirmatory focus groups and King ’s (1998) general proposal of *template analysis* for textual data, we adapted the approach for the analysis of the interview transcripts. The artifact’s DPs served as the initial coding categories.

In general, the interviewees confirmed the usefulness of the way the *image acquisition (DP1)* is performed and also acknowledged the *image processing (DP2)* to extract comparable, trustful, and helpful information. They specifically confirmed the usefulness of the vision-based approach for capturing a wide variety of different faults. More significantly, the ability to “[...] look into the detailed pictures is already of high value” (Iota) since it is easier to scope maintenance operations from component images rather than plain table entries. Additionally, the interviewees emphasized the good quality of the images as well as the improved perspective to view the PLCs and respective defects, due to the UAVs being able to fly close to the component of interest. Similarly, the functionality to automatically analyze the images for components and their faults was perceived as a major gain and precisely addressed the request of interviewee Zeta: “It would actually be quite interesting if someone or something evaluates these pictures that the drone captures and then just sends the damage.” The interviewees stressed several particular factors. First and foremost, the prevention of subjectivity was mentioned, leading to a uniformity in fault diagnosis and consequently to a flawless comparability between faults. Second, besides the presented ability to detect *insulators*,  *fittings*,  *birdnests* and *safetypins*, the interviewees assumed that several other components could be added easily. However, in more detail two participants raised doubts about the system’s ability to recognize severe incidents such as completely broken and consequently dangling insulators. Finally, six out of the nine participants indicated, without being asked, that they felt there were benefits in using an automated process to extract defective components. They specifically mentioned benefits regarding timeliness, cost, and performance in comparison to the current manual inspection methods. However, although the proposed extraction of faults generated generally positive feedback, the need to “comprehend: how did this assessment come about” (Ny) was mentioned. Consequently, both the results and the reasoning of the fault diagnosis require visualization.

### User Interface Component

Supporting MEs based on improved data and information quality requires making them accessible through a UIC. In the following, we describe the UICs’ design and evaluation.

#### Instantiation of the User Interface Component

**Fig. 7:**
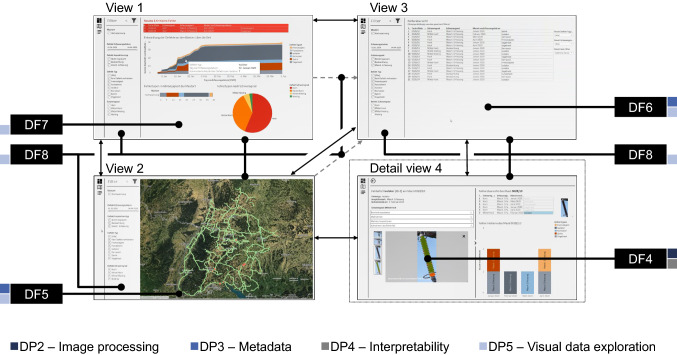
Structure of our user interface with its different views and the transitions between the views

To create a UIC that accomplishes the preliminary DRs of visualization (DR5) of the network and related defects (Shakhatreh et al. 2019), we implemented the artifact based on the inferred DPs (cf. Fig. 2 on page 15) using Tableau^6^ and Javascript. The artifact integrates two data sources: (1) UAV-captured image data (DP1) and its according metadata (DP3) as well as (2) metadata about the physical objects of the power line infrastructure (DP3) at our case company, such as geographical position or age. Information that is extracted as part of the image processing (cf. Sect. 5.2) is integrated into the artifact (DP2 and DP4). Finally, these building blocks are arranged in a meaningful way to support decision-making through visual data exploration (DP5). Figure 7 depicts the different views and their interactive links along with the respective DFs.

To satisfy DP5, the general layout of the UIC should follow the visual exploration paradigm (Shneiderman 1996) and provide overview first, allow for zoom and filter capabilities, and then accommodate details on demand. We base our UIC on four different views which emphasize different task properties in our multidimensional data and maximize the availability of explicit and latent information. View (1) provides an operational dashboard view (DF7) to get a quick and aggregated sense of the condition of the power line infrastructure. View (2) contains a geographical information system view (DF5) to find and inspect adjacent infrastructure items and faults. This allows MEs to explore both the incident location to determine maintenance order specifics as well as further incidents which can be bundled. View (3), a list-based view (DF6), enables MEs to examine a large number of faults regarding their attributes as well as to find specific faults. This may help in either bundling incidents, making sourcing decisions upon resource scarcity, or ordering replacement components. View (4) presents a fault detail view to inspect particular faults regarding the results of the fault diagnosis, including properties, specifics, and context. It consequently enables MEs to assess the faults priority, judge the skills required for the faults resolutions, and determine the affected circuit. The interactive visualization (DF8) allows MEs to directly interact with the visualizations to obtain and extract the relevant data at the right time. A persistent filter sidebar with domain-specific filters provides consistency across the first three views. While View 1 through 3 already provide different levels of zoom, the list-based view is the closest to viewing a single fault. Users are therefore able to filter subsamples of faults in View 1 as well as 2 and through interactive linking consequently invoke their display in the list-based View 3. Finally, detailed information on a particular fault identified either in View 2 or 3 can be examined. Images of the defective component are available in a gallery. To address DP4, the gallery provides the user with visual fault explanations (DF4) of the component for improved interpretability of the fault diagnosis. In particular, the detected defective component is framed by a bounding box for convenient localization. Additionally, based on the type of fault either a segmentation mask (for insulators) or a heat map (for splints) is visualized. Besides the image gallery, the user is able to expand related information showing other faults on the power tower and the fault timeline of the power tower. As a summary, a video demonstration of the user interface shows all described views in detail.^7^

#### Evaluation of the User Interface Component (EE II)

For the evaluation of our UIC, we applied a qualitative evaluation to test the proof of applicability in the real-world context and to assess the usefulness as well as efficiency. In particular we aimed to answer the evaluation question:

**EE II**: Does the instantiated UIC support MEs in making improved decisions about planning and scoping individual maintenance orders?

To answer this question, we remotely^8^ conducted nine one-on-one, confirmatory workshops with the same participants already questioned in EE I.II over the company’s collaboration platform. This confirmatory evaluation approach was chosen for two reasons. First, the flexibility of the method enabled us to adapt the procedure if necessary. Second, each user was able to individually explore and use the prototype in their accustomed work setting, which allowed the integration into the user’s working routine and ensured that the artifact and its capabilities were understood unambiguously.

For each workshop, we initially introduced the intent of the UIC. We subsequently started a screen sharing session and asked each participant to explore and use the UIC and verbalize their thoughts. Whenever appropriate, the researcher enriched the participant’s experience by providing information about the DFs. Afterwards, each participant was asked to fill out a survey based on Davis ’s (1989) technology acceptance model (TAM). Finally, the participant was asked to evaluate whether the presented artifact addresses its decisive DPs during a semi-structured interview. The question of whether each presented DF appropriately addresses its respective DPs served as the starting point. The transcripts of the workshops were analyzed in analogy to E I.II, using template analysis by King (1998).

The survey results as well as the results from our qualitative evaluation indicate that our instantiated artifact is able to support MEs in their decision-making regarding PLCs. While our TAM survey comprising the nine interviewed experts cannot claim significance, it suggests the tool’s usefulness as the perceived usefulness averaged 6.2 on a 7-point Likert scale. In accordance, the interviews revealed that the artifact would support the MEs in their everyday work by enhancing the availability of data and information of the power line infrastructure and the appropriate arrangement of the information. The confirmatory workshops therefore showed that the underlying design knowledge is suitable, useful, and effective for developing IB-DSS artifacts aimed at the vision-based maintenance of infrastructure.

In particular, the participants mentioned that the IB-DSS allows fast and convenient *visual data exploration (DP5)* while being helpful to experienced workers as well as (and especially) those in training. The interviewed experts mentioned that the artifacts’ capabilities for overview, interactive zooming, and interactive filtering are the main facilitators for convenient exploration. The interactive zooming across the multiple views makes latent information, for example staggering faults on one passage or the circumstances around a tower, visually available. Finally, the filter capabilities support finding relevant faults, as “[one] can filter out the unimportant ones” (Eta). However, six participants requested additional filters based on further metadata concerning the components in the infrastructure. While the available *metadata (DP3)* regarding towers and their identified faults was perceived as a good starting point, all participants mentioned further data which could be integrated: fault-related workflow tracking metadata as well as component-related material and reordering metadata. The participants also recognized that the visual fault explanations could mainly help them localize faults significantly faster as well as develop a thorough comprehension and understanding of the fault. Specifically, it was mentioned that the easier localization could reduce the workload and accelerate the root cause analysis. On the downside, it could hinder independent examination of the images in the long run. The image augmentations consequently provide fault *interpretability (DP4)*. Most significantly, all participants acknowledged that the IB-DSS is especially suitable for improved maintenance decision-making, as they would be able to “work more efficiently, simply work more or even combine activities” (Epsilon). In fact, besides the planning and scoping of individual maintenance orders, the improved data and information availability and quality enhance four key decision-making tasks: finding and discovering systematic faults (Epsilon, Iota, Lambda), combining maintenance orders (Eta, Epsilon, Kappa), discussing maintenance budget (My, Iota, Ny), and scoping and planning long-term restoration projects (My, Kappa).

## Discussion

In this section we depict the contributions and limitations of our work and present an outlook regarding PLC inspection and maintenance.

### Contributions

Our results imply that our instantiated IB-DSS enables maintenance engineers to make better, more informed decisions about repairing or replacing PLCs by means of improved data and information quality.

**Fig. 8:**
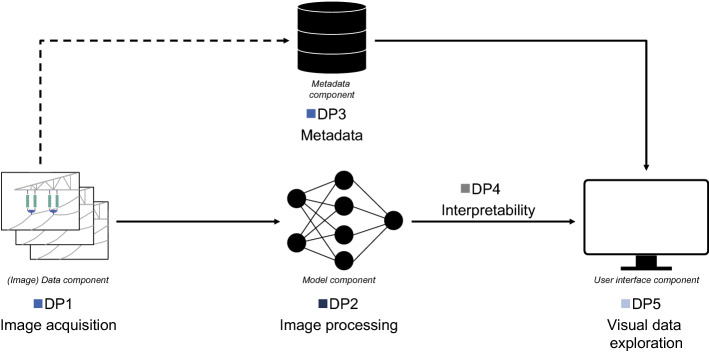
Schematic layout of the design principles of image-based decision support systems

More generally, this suggests that the rich information from uniformly acquired images extracted through deep-learning-based image processing capabilities combined with contextual information of metadata and interpretability provided by visual data exploration is a valuable solution to the information intensive context of maintenance and monitoring applications. Figure 8 depicts the schematic layout of these aspects. Consequently, we hypothesize that our derived knowledge provides a nascent design theory for the still underresearched class of IB-DSS. This design knowledge might be particularly valuable for creating automated decision support systems in information-intensive contexts where decision-makers largely rely on unstructured vision-based image data. This in turn would increase the quality of decision both in terms of efficiency and effectiveness (Kraus et al. 2020).

The schematic layout of our conceptualized design principles, as depicted in Fig. 8, therefore provides prescriptive knowledge that may serve as a blueprint (Gregor and Jones 2007) to develop similar systems for vision-based applications.

In our specific use case of PLC inspection, the proposed IB-DSS relying on UAV generated images can provide multiple benefits compared with the status quo. It can prevent accidents since hazardous inspection methods like tower-climbing are no longer necessary – as the inspection of the towers is now performed by unmanned UAVs. While no coherent numbers are available within Europe, recent reports from the US demonstrate that power line work is listed among top 10 most dangerous jobs. Each year, over 40 power line workers receive fatal injuries resulting from falling or electrocution (Schwarz and Drudi 2018). While certainly only a share of these workers die during inspection activities (rather than the repair activity itself), it is desirable to save every life possible. The non-fatal injuries amount to 1200 per year in the US (Schwarz and Drudi 2018) and the typical reasons are falling, slipping and tripping. We also expect significant reduction of injuries in this area, once automation of inspection is implemented.

Currently, the data that MEs work with are tables of compiled inspection reports with heterogeneous assessments of a distributed workforce. The standardized data acquisition and processing results in (1) more reliable and (2) more structured data. Combined with the benefits of a unified interface that provides metadata and latent, information maintenance decisions are fully comprehensible.

In total, the participants of the confirmatory workshop affirmed that the IB-DSS enhances their decision-making substantially. As mentioned by Epsilon, Theta, Kappa, and My, besides the pure planning of maintenance orders the artifact could moreover be utilized for other tasks, like the combination of maintenance orders or the planning of long-term restoration projects.

### Limitations

While meeting Gregor and Jones ’s (2007) six common criteria for design theories, our design knowledge for IB-DSS carries limitations that open opportunities for future research. Our research can only be generalized to a limited extent because it was conducted at one company in the power line infrastructure domain and focused on a selection of defect cases. While we can claim some generalization by supporting our design through kernel theories and other studies, further IB-DSS should be developed for other use cases and in other domains to extend and consolidate the design theory. Furthermore, our research lacks quantification of the effect on the field efficiency of the image processing. Quantitative studies in this regard could be conducted to benchmark the artifact’s effects in terms of performance of automated versus manual image processing.

### Future Design Activities

**Fig. 9:**
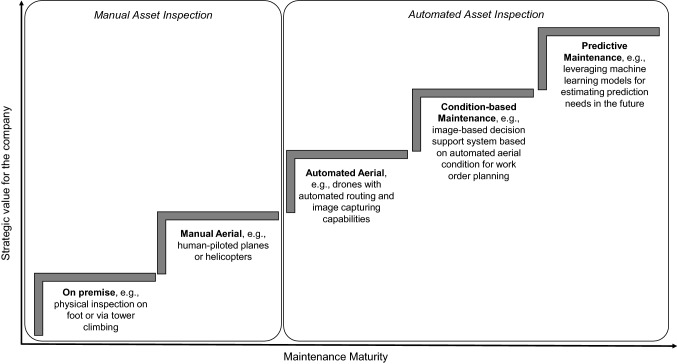
Road map towards predictive maintenance

Within our presented research, we showed novel ways to design condition-based maintenance systems. More precisely, we utilized images captured by UAVs which were subsequently automatically analyzed and included within an image-based decision support system. Figure 9 shows a possible general road map demonstrating increasing maintenance maturity, with the next evolutionary step being to use the data as well as the generated models not only as a basis for maintenance order planning, but moreover to predict maintenance needs for the (distant) future, i.e., predictive maintenance.

In regard to the practical aspects at the case company, the artifact is currently prepared for a broader implementation and deployment into the business. For these steps, the solution is containerized (Rufino et al. 2017) to allow for flexible and scalable applications. An expert team analyzes the different possibilities of automated UAV routing (Avellar et al. 2015) to allow for a continuous and correct collection of the required data. Meanwhile, experts are being educated on the possibilities of integrating the tool into their current day-to-day processes, supported by an expert for change management of industrial business processes (Bokrantz et al. 2020). One remaining challenge is the aspect of data storage and management, e.g., within a data warehouse. On the basis of the required data volume shown in Table 5 on page 20, we estimate a total volume of images for a one-time acquisition of the complete network of our case company of 9 TB. How often this data has to be refreshed and how precisely it is stored (e.g., hybrid cloud) needs to be discussed for future iterations of the artifact.

In a broader context, the automated inspection of PLCs will be an important, yet only intermediate step for PLC maintenance in the future. The image data used in this work can be combined with multiple additional data sources such as weather and location characteristics (e.g., sun exposure and topology). The inclusion of additional sources of information can enable an accurate prediction of future maintenance needs which further facilitate effective planning and resource utilization.

## Conclusion

Planning and preparing maintenance orders in power line maintenance is a challenging task for maintenance engineers, as they must rely on human-created, heterogeneous, and largely unstructured information. These characteristics make the process both time-intensive and costly, which can adversely affect the continuous supply of electricity. As most research on power line maintenance focuses on automated inspection through UAV-captured images and deep learning, there is an apparent gap in literature for transferring the acquired data into maintenance decision-making.

Following the design science research guidelines, we designed, developed, and evaluated an artifact to address this research gap. Initially, we rigorously analyzed the challenges in power line maintenance. Building on these, we conceptualized design principles for an image-based decision support system that integrates the capabilities of deep learning to extract faulty components from a set of captured images and appropriately presents the information to relevant users. Accordingly, we implement our design principles in an exemplary artifact. The evaluation using a technical experiment as well as two qualitative evaluation episodes with long-standing experts indicates the utility of our design knowledge and can therefore inform future system designs of similar nature.

## Supplementary Information

Below is the link to the electronic supplementary material.

Supplementary file 1 (PDF 197 KB)

Supplementary file 2 (MP4 47527 KB)

## Footnotes

1 Note that we will use the term image processing and computer vision interchangeably, as there is no common agreement between the boundaries of the two terms (Gonzalez and Woods 2018).

2 For a general introduction into machine learning and deep learning, we refer the interested reader to Janiesch et al. (2021).

3 Version 1.15.

4 In particular, the tensorflow object detection API.

5 Version 2.3.1.

6 https://www.tableau.com/.

7 https://youtu.be/Y3oIJghtRT4.

8 Due to COVID-19.
