# Supplementary material for: Design Knowledge for Deep-Learning-Enabled Image-Based Decision Support Systems: Evidence From Power Line Maintenance Decision-Making
Source: Bus Inf Syst Eng. 2022 Apr 1;64(6):707–28. doi: 10.1007/s12599-022-00745-z (PMC8973684; doi:10.1007/s12599-022-00745-z)
Supplement: Supplementary file 1 — (PDF 197 KB) [file 12599_2022_745_MOESM1_ESM.pdf]

Design Knowledge for Deep-Learning-Enabled Image-based Decision Support Systems – Evidence from Power Line Maintenance Decision-Making

Julius Peter Landwehr, Niklas Kühl, Jannis Walk, Mario Gnädig

Business & Information Systems Engineering (2022)

Appendix (available online via http://link.springer.com)

Appendix

A1 Challenges of Power Line Maintenance

Challenge C4 attributes to organizational or administrative levels of introducing novel solutions which include the consideration of the exact purpose of the infrastructure (C4.1), the cost associated to their inspection (C4.2), and specific challenges that come with the culture, digitization maturity, and capabilities of an organization (C4.3). Moreover, the challenge C5 addresses the fact that power lines are considered as critical infrastructure which's operations, inspection, and maintenance is strictly regulated. Another challenge that applies to generally all infrastructure related inspections is of environmental kind (C6). Environmental challenges includes limitations in the maintenance of power lines due to weather conditions and general seasonal circumstances.

Table 7.1 Further challenges in the maintenance of power lines based on expert interviews and a structured literature review

ID	Challenge	Subchallenge	Source
**C4**	Organizational challenges	C4.1—Significance of uninterrupted power supply	Pagnano et al. (2013); Nguyen et al. (2018); Prasad et al. (2016) Li and Wang (2019); Matikainen et al. (2016); Toussaint et al. (2009); Katrasnik et al. (2010); Seok and Kim (2016); Beta
		C4.2—Scale of inspection cost	Pagnano et al. (2013); Nguyen et al. (2018); Mirallès et al. (2014); Prasad et al. (2016); Deng et al. (2014); Jones (2005); Aggarwal et al. (2000) Li and Wang (2019); Takaya et al. (2019); Pernebayeva and James (2020); Matikainen et al. (2016); Katrasnik et al. (2010); Seok and Kim (2016); Huang et al. (2018); Ostendorp (2000); Alpha; Beta; Gamma; Delta
		C4.3—Company-specific challenges	Alpha; Gamma; Delta
**C5**	Regulatory requirements	C5.1—Compliance with regulations	Pagnano et al. (2013); Prasad et al. (2016); Jones (2005); Takaya et al. (2019); Matikainen et al. (2016); Toussaint et al. (2009); Gamma
**C6**	Impact of environmental conditions	C6.1—Dependence on seasonal circumstances	Delta
		C6.2—Dependence on climatic conditions	Nguyen et al. (2018); Pernebayeva and James (2020); Seok and Kim (2016); Homma et al. (2017); Beta

A2 Supervised Machine Learning Report Card based on Kühl et al. (2021)

General Information	Problem statement	Detect objects of power line components (insulator, fitting, safety pin, birdnest) from an input image and classify whether the safety pin components are intact or defect.	
	Data gathering	The proprietary data set originates from the application case company Netze BW, a distribution system operator in Southern Germany. We harnessed UAVs to capture images of their high voltage power lines as part of a technology driven proof of concept.	
	Data distribution	After annotation of the images of the proprietary data set it contains (BB = Bounding Box):	
		Insulator (BB)	1,424
		fitting_top (BB)	1,073
		fitting_bottom (BB)	1,438
		Birdnests (BB)	61
		Safety Pins (BB)	(3,692 intact/1,494 defect) 5,186
	Data quality	High-quality images with a resolution of 5280x3956 pixels. Bounding boxes and labels generated by researchers who had received instructions and feedback from field experts.	
	Data preprocessing methods	Rescaling (1/255)	
	Performance Estimation		
Object Detection Task – General Information	Parameter optimization	None	
	Data split	Training data set 80%, Evaluation data set 20% *To increase the evaluation's validity, images captured at one tower were held out from the random split and solely utilized for the evaluation dataset, while maintaining the split ratio.*	
	Sampling/ Data augmentation	Random brightness adjustment	
	Performance metric	mean average precision (mAP) (Rafael Padilla & da Silva 2020)	
Faster R-CNN	Algorithm Parameters	CNN backbone	ResNet-50
		Early stopping patience (on validation loss)	100
		Optimizer	SGD (learning rate 0.0003 and 0.9 momentum)
		Batch size	1
		Performance evaluation	0.7510
SSD	Algorithm Parameters	CNN backbone	ResNet-50
		Early stopping patience (on validation loss)	100
		Optimizer	SGD (learning rate 0.001 and 0.9 momentum)
		Batch size	64
		Performance evaluation	0.7718

<table>
<tr><td rowspan="5">Classification Task – General Information</td><td>Parameter optimization</td><td>Yes</td><td>Search space
Search algorithm</td><td>cf. Table 1
Grid search</td></tr>
<tr><td>Data Split</td><td colspan="3">10% Hold out set
90% train and validation set with 3-fold cross validation</td></tr>
<tr><td>Sampling/ Data augmentation</td><td colspan="3">- Average blur [0,11]
- Brightness range [0.2,1.5]
Height shift range [0.1]
- Width shift range [0.1]
- Horizontal flip: true
- Vertical flip: true</td></tr>
<tr><td>Performance metric</td><td colspan="3">Weighted precision, weighted recall, and weighted F1-score (Pedregosa et al. 2011) to account for class imbalance</td></tr>
<tr><td colspan="4"></td></tr>
<tr><td rowspan="7">ResNet -50</td><td rowspan="6">Final algorithm parameters</td><td colspan="2">Dense layers</td><td>(512, 512)</td></tr>
<tr><td colspan="2">Unfrozen layers</td><td>3</td></tr>
<tr><td colspan="2">Dropout rate</td><td>0.1</td></tr>
<tr><td colspan="2">Early stopping patience (on validation loss)</td><td>30</td></tr>
<tr><td colspan="2">Optimizer</td><td>Adam (learning rate 0.0005)</td></tr>
<tr><td colspan="2">Batch size</td><td>32</td></tr>
<tr><td>Performance evaluation</td><td colspan="3">AUROC: 0.8080
Weighted precision: 0.76
Weighted recall: 0.76
Weighted F1-score: 0.71</td></tr>
<tr><td rowspan="7">VGG16</td><td rowspan="6">Final algorithm parameters</td><td colspan="2">Dense layers</td><td>(512, 512)</td></tr>
<tr><td colspan="2">Unfrozen layers</td><td>8</td></tr>
<tr><td colspan="2">Dropout rate</td><td>0.1</td></tr>
<tr><td colspan="2">Early stopping patience (on validation loss)</td><td>30</td></tr>
<tr><td colspan="2">Optimizer</td><td>Adam (learning rate 0.0005)</td></tr>
<tr><td colspan="2">Batch size</td><td>32</td></tr>
<tr><td>Performance evaluation</td><td colspan="3">AUROC: 0.8114
Weighted precision: 0.80
Weighted recall: 0.80
Weighted F1-score: 0.78</td></tr>
</table>

Table 7.2 Parameter set options for the training of convolutional neural network for both ResNet-50 and VGG16

Parameters	Dense layers	Unfrozen layers	Optimizer	Learning rate	Batch size	Dropout rate
Ranges	((512, 512)<br>(512, 1024)<br>(512, 2046)<br>(1024, 1024)<br>(1024, 2046)<br>(2046, 2046))	[3, 8]	Adam, SGD	(0.0005,<br>0.001,<br>0.0015,<br>0.002)	(32, 64, 128)	[0, .6]

A3 Questionnaire (translated from German to English)

1. *General introduction:*
   How was the "application" of the the Image-based Decision Support System (IB-DSS) for you?
   - Would you use the IB-DSS in practice?
   - What worked particularly well?
   - What did not work well?
   - What possibilities result from the application of the IB-DSS?
   - What problems could occur during the application?

   Model component:

2. **Design Principle 1:**
   Unmanned aerial vehicle (UAV) & RGB images
   Was the quality of the RGB images of the UAVs sufficient and do they enable a good overview over the most important properties / the condition of the infrastructure?
   - Were you lacking important images / information / data?
   - Were the images from the UAVs well inspectable?
3. **Design Principle 2:**
   Deep Learning for Computer Vision
   Does the usage of machine / deep learning enable additional, helpful information (e.g., severity, defect type, etc.)?
   - What chances and risks arise through the IB-DSS?
   - What strengths and weaknesses does the IB-DSS possess regarding the recognition of faulty / defect components?

   User Interface Component:

4. **Design Principle 3:**
   Interpretability
   How did the accentuation of the condition of components influence the interpretability? Was it a good assistance to understand the result?
   - Is the IB-DSS a good tool to comprehend the condition of a component?
   - What are the advantages of the visualization?
   - What are the disadvantages of the visualization?
   - What problems can occur due to the visualization?
5. **Design Principle 4:**
   Exploratory (Data) Visualization
   Does the IB-DSS enable an investigation / exploration of the data? Does it facilitate to gain information about the condition of power lines and a corresponding overview?
   - What are the strengths and weaknesses of the visualisation of the condition data?
   - How important is the availability of the data?

  - What long-term chances and risks do you see regarding the maintenance process?
6. **Finalization**
   Anything else you would like to share with me?
   - Where do you see room for improvement?
   - Is there any other feedback you would like to share?
